# Multifocal Hepatic Angiosarcoma with Atypical Presentation: Case Report and Literature Review

**DOI:** 10.1007/s12029-020-00504-x

**Published:** 2020-09-07

**Authors:** Stefano Marletta, Enrico Cavallo, Serena Ammendola, Lavinia Stefanizzi, Maria Gaia Mastrosimini, Mirko D’Onofrio, Matteo Brunelli, Anna Caliò, Sara Pecori, Andrea Dalbeni, Andrea Ruzzenente, Paola Capelli

**Affiliations:** 1 Department of Diagnostics and Public Health, Section of Pathology, University of Verona, P.le L.A. Scuro n. 10, 37134 Verona, Italy; 2 Department of Diagnostics and Public Health, Section of Department of Radiology, University of Verona, Verona, Italy; 3 Department of Pathology and Diagnostics, University and Hospital Trust of Verona, Verona, Italy; 4 Department of Medicine, Section of General Medicine and Hypertension Unit & Liver Unit, University of Verona, Verona, Italy; 5 Division of General and Hepatobiliary Surgery, Department of Surgical Sciences, Dentistry, Gynecology and Pediatrics, University of Verona, Verona, Italy

## Introduction

Hepatic angiosarcoma (HAS) is a malignant mesenchymal neoplasm composed of highly atypical, pleomorphic endothelial cells, which diffusely grow along liver sinusoids and other preformed vascular channels, especially at the periphery of the tumor, replacing normal endothelial cells. More solid areas, resembling sarcomas, can be found [1]. Far less common than hepatocarcinoma, representing less than the 2% of all the primary malignant hepatic tumors, HAS is still the most frequent primary malignant mesenchymal neoplasm of the liver. Although most of HAS do not carry a specific etiology, several chemical carcinogens, including vinyl chloride monomer (VCM), Thorotrast (thorium dioxide), and arsenic, have been listed as being responsible for occupational cases. VCM is a colorless, sweet-smelling gas primary used in the production of polyvinyl chloride (PVC), whose resins are almost ubiquitously present in building materials, construction, and home furnishings. VCM is now classified as an International Agency for Research on Cancer (IARC) Group 1 carcinogen, due to the correlation between HAS and its occupational exposure [2]: initially described in three workers at a VCM polymerization plant in Kentucky in 1974 [3], several other HAS occupational cases have been reported worldwide in the following decades, all linked to workers’ exposure to VCM [4, 5].

Because HAS is an extremely rare tumor, both radiological and histological diagnosis could be really challenging and their integration with clinical data and history has a key role in reaching the correct diagnosis.

Herein the case of a primary multifocal HAS is described, with initially misleading unspecific radiological findings. Suggesting the biopsy a malignant vascular lesion of the liver, an autopsy was performed, which confirmed the diagnosis of HAS.

## Case Report

A 73-year-old man with a history of hypertension and congestive cardiac failure was admitted at our hospital for progressive jaundice of unknown etiology. During prior hospitalizations multiple hepatic nodular lesions of uncertain nature had been reported at various imaging exams; furthermore, two ultrasound-guided liver biopsies had already been formerly performed, leading to a diagnosis of nodular regenerative hyperplasia of uncertain interpretation.

After admission to our hospital, laboratory tests revealed increased direct bilirubin levels (9.59 mg/dl), while liver enzymes were within the recognized normal ranges. Moreover, search for autoantibodies and serologic tests for hepatotropic viruses were negative and no anomaly was noted for blood iron parameters.

**Fig. 1:**
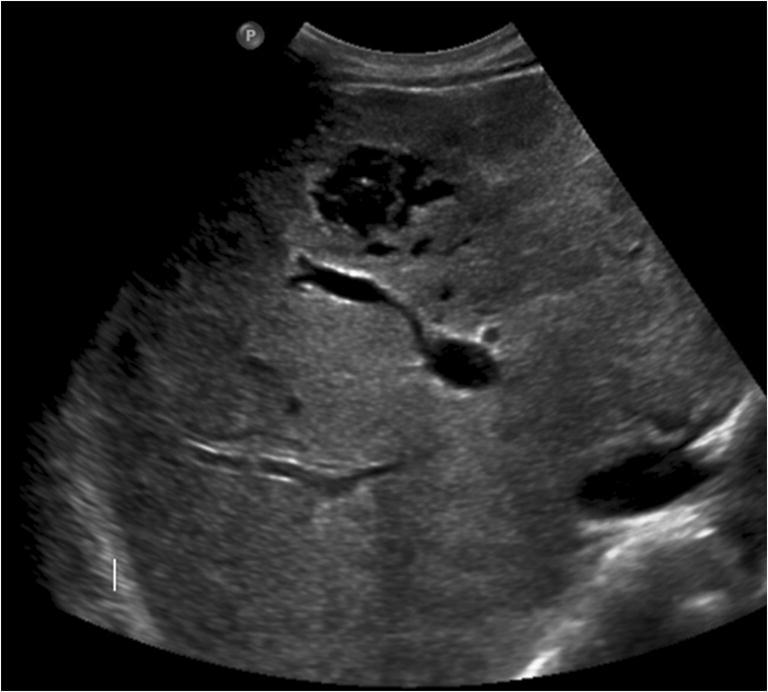
Ultrasound: multiple focal liver lesions appearing markedly inhomogeneous. Same lesions show fluid component

**Fig. 2:**
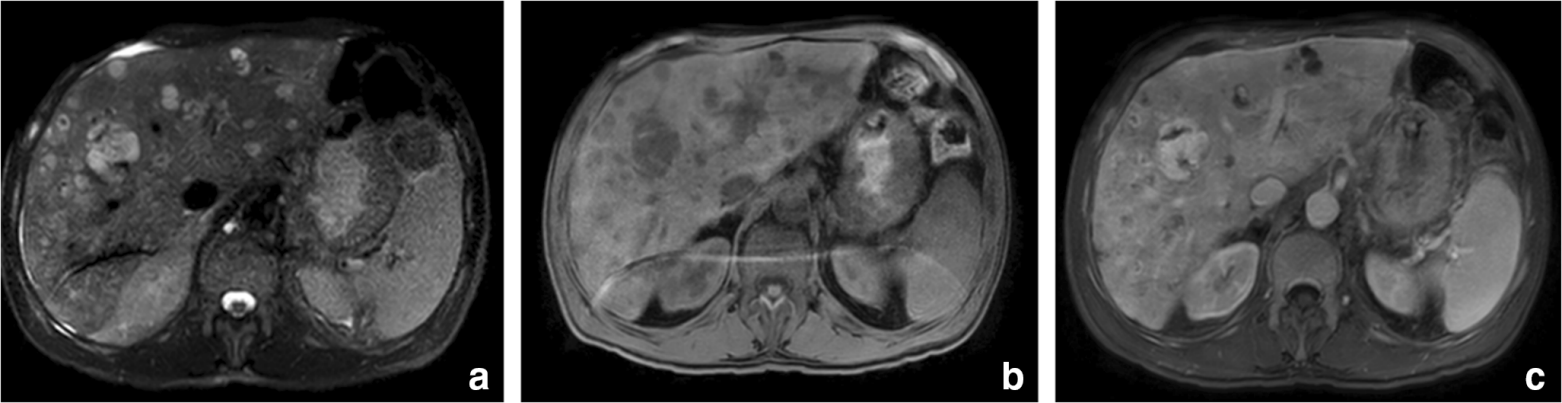
MRI: multiple focal liver lesions appearing hyperintense on T2 (**a**) and hypointense on T1 (**b**). After contrast media injection the lesions show fill in appearing hyperintense on venous phase (**c**)

Abdominal contrast-enhanced US revealed multiple hyperechoic, anaechoic, and isoechoic hepatic nodules (Fig. 1). Abdominal contrast-enhancement computerized tomography (CT) and magnetic resonance imaging (MRI) revealed increased liver volume, with left lobe hypertrophy and heterogeneous appearance of the parenchyma: multiple confluent lesions with numerous blanks were detected, respectively showing low-density attenuation at CT and heterogeneous low intensity on T2-weighted imaging (WI) and heterogeneous high intensity on T1-weighted imaging (WI) at MRI. After contrast administration, a well-developed vascularization of the blanks was noted with both the methods, namely, with inhomogeneous and incomplete enhancement on T2-weighted imaging (WI) on MRI (Fig. 2a–c). These atypical radiological findings were utterly unspecific for any particular disease: not only for their diffuse and bilobar massive spreading throughout the liver but also for the involvement of both central and peripheral areas of the hepatic parenchyma. The blank characteristics suggested the presence of vascular spaces, somehow recalling those observed in benign lesions such as hepatic peliosis.

After multidisciplinary team discussion, a laparoscopic liver biopsy was performed. The histological findings were suspicious for a vascular lesion but, before a definitive diagnosis could be made, 3 days after the procedure the patient’s conditions rapidly worsened, evolving in acute respiratory distress syndrome (ARDS) and severe liver failure. Ultimately, the patient developed hepato-renal syndrome and died.

**Fig. 3:**
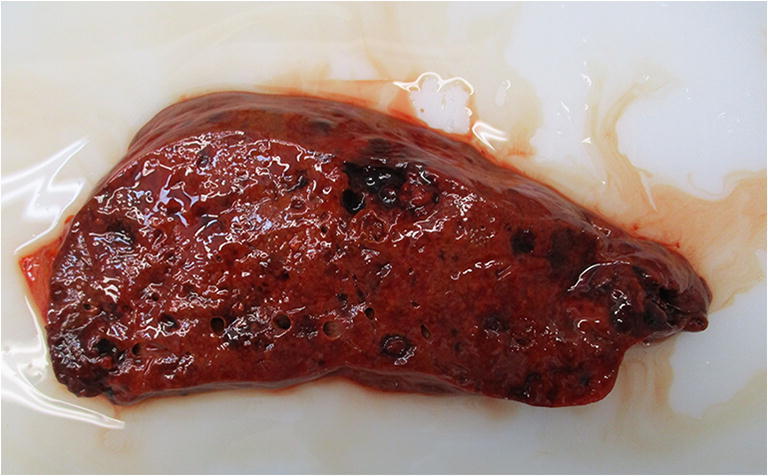
Blood filled cavities and gray-brown spongy areas surrounded by normal parenchyma at cut examination

In order to find the cause of the sudden deterioration of the liver function, an autopsy was requested. Grossly, the liver had an irregular surface and revealed blood filled cavities and gray-brownish spongy areas surrounded by recognizable residual normal hepatic parenchyma at cut examination (Fig. 3). No other lesions were found in any other organ.

Twenty-four tissue samples were taken from the liver and then fixed in 10% formalin solution and embedded in paraffin blocks. Four-micrometer-thick sections were obtained and stained with hematoxylin and eosin for histopathological examination. Immunohistochemical staining for CD 31 (clone JC/70A, DAKO, dilution 1:50), CD34 (clone QBEND/10, Novacastra, dilution 1:200), and ERG (clone EPR3864, Abcam, dilution 1:200) was performed.

**Fig. 4:**
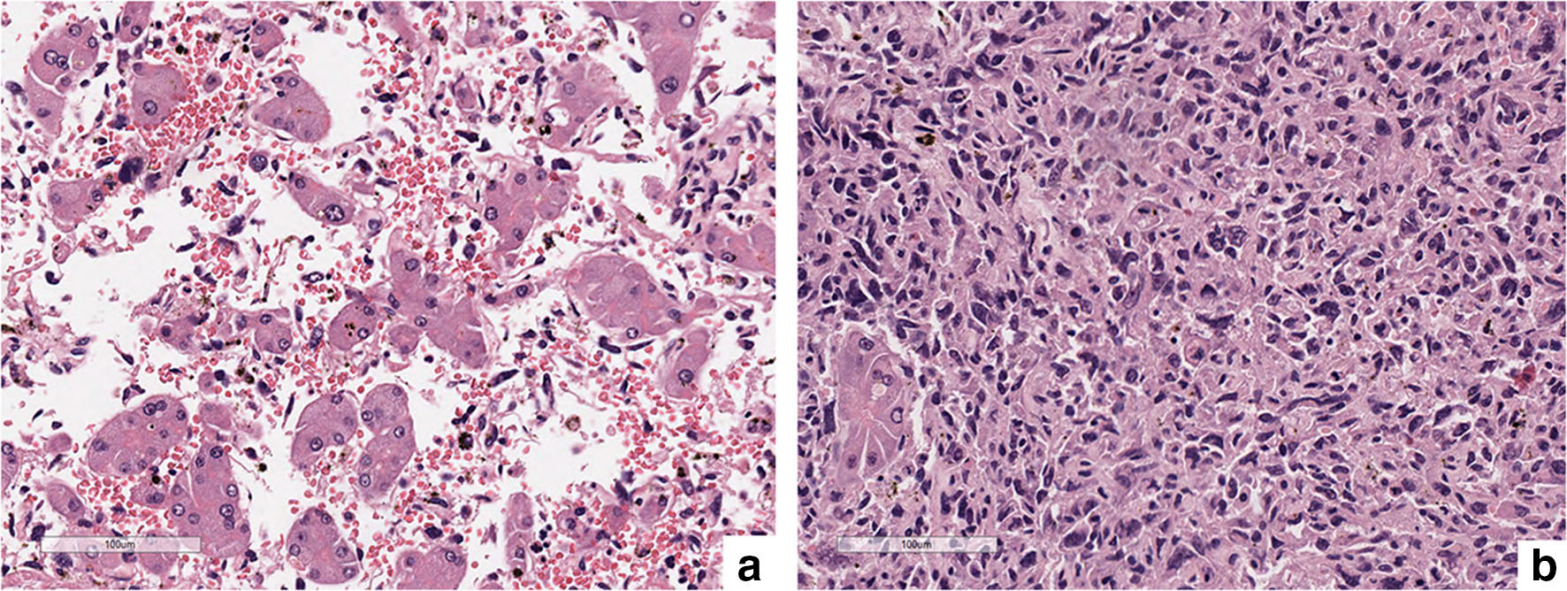
Highly atypical endothelial cells covering cavernous space (**a**); nodular area showing dilated sinusoids filled with atypical neoplastic spindle cells (**b**)

**Fig. 5:**
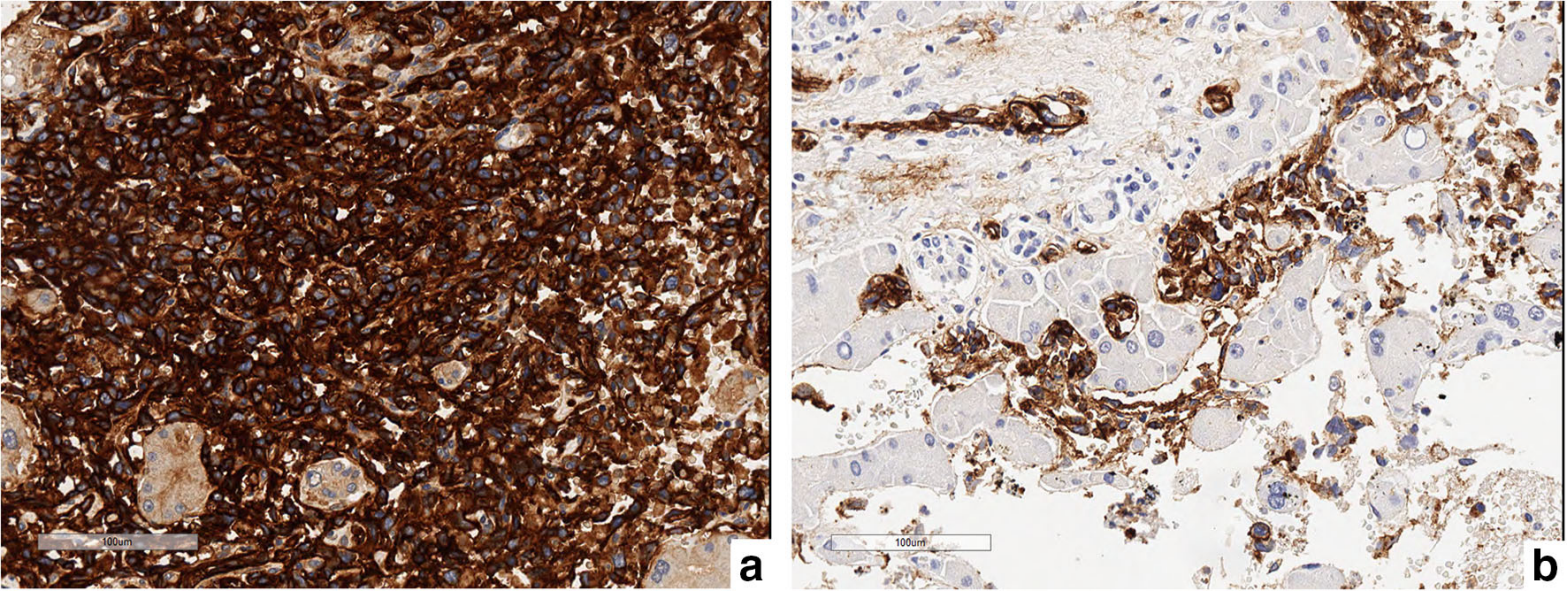
Nodular area with plenty of CD31 positive neoplastic cells (**a**); CD34 positivity of neoplastic spindle cells in a less cellular area (**b**)

Microscopically, multiple cavernous spaces covered by highly atypical endothelial cells were observed (Fig. 4a), along with nodular areas made up of spindle cells surrounded by dilated sinusoids filled with neoplastic cells were seen (Fig. 4b). The atypical cells showed diffuse and strong positivity for endothelial markers, such as CD-31 (Fig. 5a), CD-34 (Fig. 5b), and ERG.

A chronic occupational vinyl chloride exposure was retrospectively discovered. Based on these clinical data and the morphologic features, the diagnosis of multifocal HAS was established.

## Discussion

Hepatic angiosarcoma (HAS) is a very aggressive malignant mesenchymal tumor arising from liver endothelial cells composed of atypical, plump, pleomorphic cells with hyperchromatic nuclei and high mitotic rate. Although solid areas of sarcomatous-looking neoplastic cells, admixed with giant multinucleated cells, can be found, HAS’s neoplastic cells tend to grow mainly within preexisting vascular channels, especially along hepatic sinusoids, without gathering in a distinct and compact mass (diffuse, cavernous type). Tumor cells show an endothelial phenotype, staining commonly positive for CD31, CD34, ERG, and FVIII; a small percentage of cases is reported to express pan-cytokeratins. HAS is frequently a multifocal disease, with the liver’s parenchyma not uncommonly almost completely wiped out by the tumor.

HAS is a rare entity, accounting for less than the 2% of all the primary malignant hepatic neoplasms, with about 200 cases worldwide diagnosed every year. Although a broad part of all HASs do not recognize any specific etiology, some chemical carcinogens have been claimed to be responsible for occupational cases, above all vinyl chloride monomer (VCM), Thorotrast (thorium dioxide), and arsenic [1]. VCM is a colorless, sweet-smelling organochloride gas primarily used in the production of polyvinyl chloride (PVC). PVC distribution is fairly ubiquitous in our society, as its resins are found in building materials, constructions, and home furnishings. Vinyl chloride toxicity is mainly linked to inhalation of its highly reactive epoxide chloroethyelene oxide through occupational exposure during the polymerization of VCM into PVC. Hepatolienal damage by vinyl chloride was demonstrated in 1949, and several series of VCM-related occupational HASs have been reported in the following decades [4, 5], with the first case in 1974 [3]. Therefore, VCM has been classified as an International Agency for Research on Cancer (IARC) Group 1 carcinogen. However, a long and chronic exposure to VCM is thought to be required for hepatic angiosarcoma HAS to arise, with an estimated latency period of 10–40 years (compared to 60 years or more in non-occupational cases) [6]. Apart from HAS, chronic occupational VCM exposure has also been linked to hand skin thickening, Reynaud phenomenon and acroosteolysis [2]. Cirrhosis, hepatocellular carcinoma, brain cancer, and lung cancer have been reported in these workers as well, although such correlations are not as strong as the formers [7]. Thorotrast is a radiocontrast used in clinical practice for 30 years during the 1930s and then retired for being accustomed with several malignancies, including HAS: namely, as for the liver, it is initially stored in Kuppfer cells and then tends to spill over into the periportal space and in the walls of terminal hepatic venules, where it can induce endothelial damage and mutagenic effects. Despite chronic arsenic exposure is more commonly linked to hepatomegaly, fibrosis, and cirrhosis, a 10–40-year prolonged environmental exposition has also been related to HAS [1].

HAS’s diagnosis may sometimes be extremely challenging, potentially representing both a radiological and histological pitfall. Namely, radiologists can face difficulties in identifying HAS for several reasons. Firstly, due to its rarity, physicians are not so likely to have to deal with these tumors in their daily routine. Secondly, HASs’ imaging findings are generally not specific, with extremely variable radiological patterns appreciated, mainly regarding the number of neoplastic nodules and the prevalence of necrotic or hemorrhagic areas within them [1, 6, 8–11]. Therefore, they are not uncommonly misdiagnosed as other liver diseases, both benign, like cavernous hemangioma [11, 12] or another vascular process, possibly resembling peliosis [13] (like the current case), and malignant, such as atypical hepatocarcinoma nodules [6] or hypervascular metastatic disease [12]. On the other hand, at gross examination HASs usually display a quite characteristic growth pattern, commonly affecting both lobes of the liver or the entire organ: multiple sponge-like hemorrhagic areas either alternating with solid grayish-white nodules or both, surrounded by apparently normal liver parenchyma [14]. As far as histology is concerned, when dealing with biopsied material from areas with a diffuse growth pattern getting to a proper pathological diagnosis could be really difficult, even with ancillary immunohistochemical techniques. Furthermore, severe hemorrhages following biopsy procedures have been reported, especially with tru-cut ones, eventually leading to death [1]. Hence, regarding all the above mentioned radiological and histological issues, our case clearly portraits how treacherous and ambiguous the presentation of HAS can be, potentially misleading the diagnosis: the diffuse and massive involvement of the liver by multiple lesions both centrally and peripherally located, with unspecific radiological features, unusual for any of the lesions most commonly encountered during daily practice, strongly contrasted with the fairly typical macroscopic and histological pathological findings observed.

Therefore, finally, for all the reported reasons a thorough evaluation of the patient’s clinical data and history and its integration with imaging and pathological findings it is mandatory for correctly assessing the diagnosis of HAS.

## Conclusions

Hepatic angiosarcoma is the most common primary malignant mesenchymal neoplasm of the liver, which has been linked to chronic occupational exposure (10–40 years) to different chemical carcinogens, such as vinyl chloride monomer, Thorotrast, and arsenic. Due to its extremely aggressive clinical behavior, which generally causes patients to die within a very short time, it is therefore mandatory to promptly reach the proper diagnosis. However, for several reasons HAS represents a potential radiological and histological pitfall. Firstly, physicians are not so likely to deal with these tumors due to their rarity, both for lowered permissible exposure limit and for more accurate worker surveillance programs. Secondly, unspecific imaging findings have been repeatedly reported, suggesting various different hepatic diseases, either of a benign, malignant or, as in our case, unknown nature. Finally, as neoplastic cells commonly use to grow singularly along sinusoidal spaces, without forming a distinct mass, histological evaluation of bioptic material could be incredibly tough work. Therefore, integration of pathological and radiological findings with patients’ clinical history is the key for avoiding wrong interpretations and reaching the correct diagnosis.

The case reported perfectly depicts all the obstacles that physicians might encounter in diagnosing such a rare and fatal condition. In fact, the initial radiological picture was that of an unspecific disease, probably vascular in origin: a proper definitive diagnosis of hepatic angiosarcoma could be only assessed following autopsy evaluation and its integration with the retrospective discovery of a chronic occupational vinyl chloride exposure.

## Abbreviations

HAS: Hepatic angiosarcoma
VCM: Vinyl chloride monomer
PVC: Polyvinyl chloride
